# Synergistic Probe Combining Lysosome Anchoring and Tumor Defense System Targeting for Precise Tumor Visualization

**DOI:** 10.1002/advs.75664

**Published:** 2026-05-13

**Authors:** Yang Shen, Jiawei Li, Ruixi Yi, Ling Shi, Wei Li, Sulai Liu, Lin Yuan

**Affiliations:** ^1^ Department of Hepatobiliary Surgery Central Laboratory Hunan Provincial People's Hospital (The First Affiliated Hospital of Hunan Normal University) Changsha P. R. China; ^2^ State Key Laboratory of Chemo and Biosensing College of Chemistry and Chemical Engineering Hunan University Changsha P. R. China; ^3^ School of Chemistry and Molecular Engineering Nanjing Tech University Nanjing P. R. China

**Keywords:** activatable fluorescent probe, high‐precision imaging, lysosome, molecular imaging, tumor imaging

## Abstract

Precise tumor imaging is essential for accurate intraoperative decision‐making, thereby directly influencing patient prognosis. Optical molecular probes enabling non‐invasive, dynamic assessment of cancerous lesions are clinically crucial. However, current optical molecular probes face challenges in dodging false‐positive and false‐negative signals at once during imaging, limiting their clinical diagnostic use. Here, we introduce a class of lysosome‐targeted, activatable optical probes (Dx‐NH_2_), leveraging the increased lysosomal abundance during tumor metabolic reprogramming. Lysosomal protonation retains probes for better single‐cell resolution and fewer false‐negative signals. To enable more high‐precision tumor imaging, we synthesized probe CN‐D‐GGT from CN‐D‐NH_2_ by introducing a γ‐glutamyltransferase substrate, following our group's prior offensive and defensive integration strategy. Due to its smart design, CN‐D‐GGT, upon activation, shows marked changes in fluorescence and photoacoustic signals, enabling its application for multi‐modal imaging. It also has high specificity, distinguishing cancer cells in co‐culture (∼6‐fold) and clearly differentiating tumors from normal and inflamed tissues (T/N or T/I signal ratios > 3.5). Importantly, it can also differentiate cancerous from adjacent tissues in clinical samples. This work has developed a probe that can accurately light tumors in complex pathological environments, with the potential to assist in intraoperative resection decision‐making, avoid excessive or missed resection.

## Graphical abstract

A new generation of high‐precision fluorescent probes based on the lysosomal characteristics in tumor metabolic reprogramming and overexpressed tumor markers. The probe (CN‐D‐GGT) selectively label cancer cells in co‐culture systems and distinguish tumor lesions from inflamedand normal tissues in vivo, enabling precise tumor imaging.

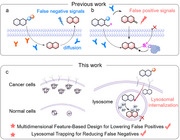

## 1 Introduction

As one of the most lethal global health burdens, cancer requires high‐precision diagnostic tools to assist clinical operations, aiming to simultaneously reduce recurrence rates and enhance patient quality of life [1]. Optical molecular probes have been used in the clinical diagnosis of cancer by taking advantage of their non‐invasiveness, high sensitivity, and high selectivity [2, 3, 4, 5, 6]. However, the current repertoire of optical molecular probes for precise tumor imaging remains limited. Two major challenges are off‐target activation, which produces false‐positive signals, and post‐activation diffusion of the fluorophore, which can result in false‐negative imaging outcomes.

**SCHEME 1:**
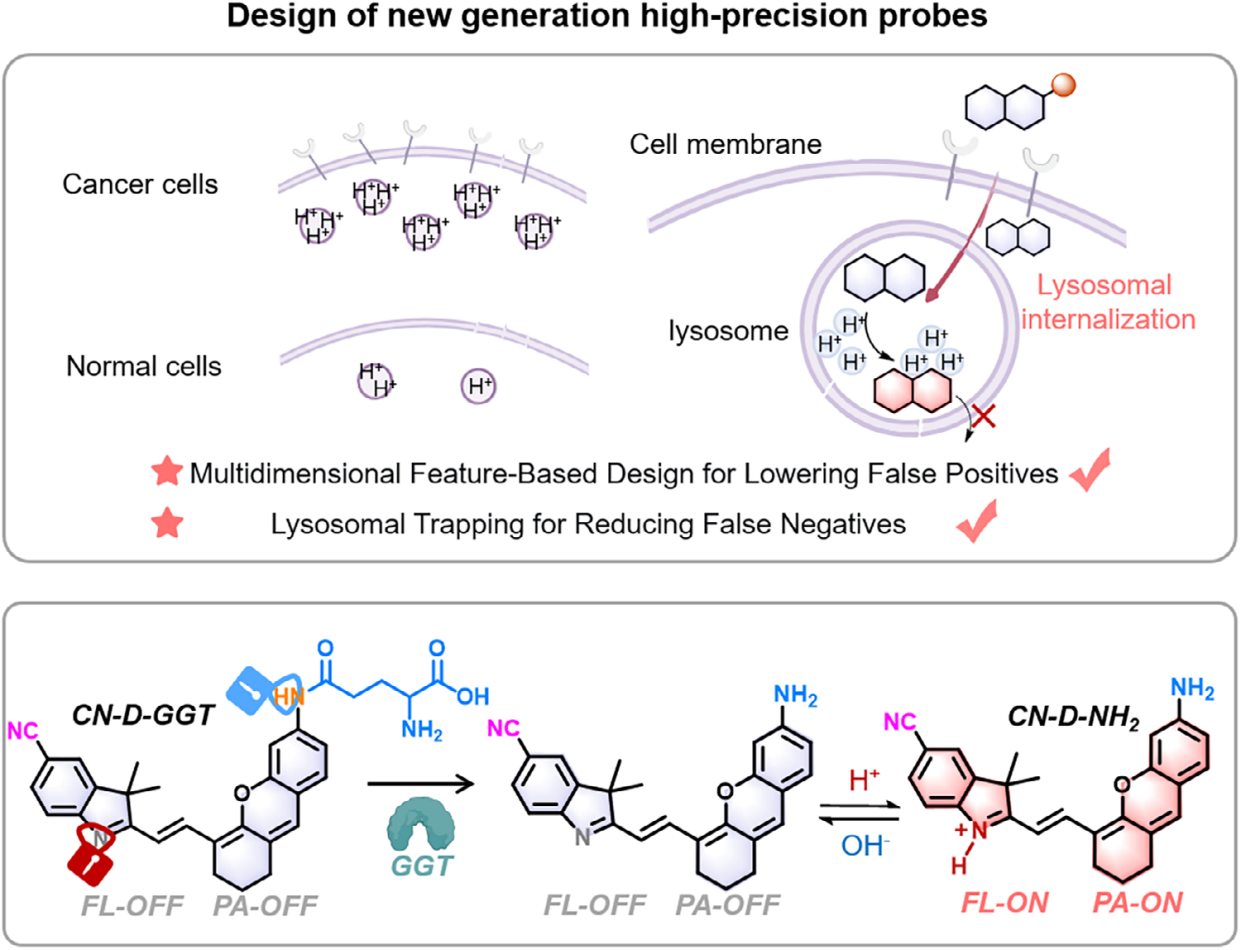
(a) Schematic illustration of the false‐negative signals generated by traditional dual‐locked probes owing to fluorophore diffusion after activation. (b) Schematic illustration of traditional functionalized probes undergoing premature activation and generating false‐positive signals. (c) Schematic illustration of the simultaneous reduction of false‐positive and false‐negative signals in tumor imaging by our probe.

To reduce nonspecific probe activation, the researchers developed a dual‐lock molecular design strategy that incorporates multiple tumor microenvironment biomarkers, thereby markedly improving imaging specificity and the signal‐to‐noise ratio in tumor imaging [7, 8]. However, a fundamental limitation of these probes lies in the diffusion of the activated fluorophore away from the lesion site, which can lead to misleading signals [9]. (Scheme 1a). To minimize imaging artifacts caused by fluorophore diffusion, a quinone methide precursor was incorporated into the probe scaffold, thereby enabling rapid covalent trapping by nearby nucleophilic proteins and achieving single‐cell‐resolution imaging [10, 11, 12]. Furthermore, increasing the polarity of the fluorophore generated upon probe activation can also enhance intracellular retention [13]. Nevertheless, these strategies often require extensive structural modification of the fluorophore, while offering only negligible improvement in activation specificity (Scheme 1b). Thus, the development of novel molecular probes capable of simultaneously overcoming the dual challenges of nonspecific activation and fluorophore diffusion holds significant research value for achieving precise tumor imaging.

Metabolic reprogramming in cancer cells promotes enhanced glucose uptake and intracellular lactate accumulation, thereby supporting the biosynthetic demands of rapid tumor growth [14, 15]. Concomitantly, dissociation of the accumulated lactate releases H^+^, which are then extruded via transporter‐mediated efflux, thereby establishing a mildly acidic extracellular microenvironment (pH_e_ = 6.5–6.8) [16, 17, 18]. Furthermore, cancer cells exhibit an increased abundance of lysosomes, which is closely linked to the proliferative and invasive requirements of their malignant phenotype [19]. Therefore, lysosomes can serve as cancer‐specific targets for developing highly specific probes. Notably, the acidic lysosomal environment can protonate weakly basic molecules, increase their polarity, and thereby enhance their intracellular retention [20]. This property provides a valuable basis for the rational design of probes with reduced off‐target effects (Scheme 1c). In summary, lysosomes not only represent cancer‐relevant biomarkers but also provide a unique microenvironment for fluorophore capture and retention. These dual characteristics of lysosomes offer a novel strategy for the development of highly precise tumor‐specific probes.

To optimize the photophysical properties, we engineered the classical hemicyanine scaffold [21] by replacing the quaternary ammonium moiety with an indole unit as a mild electron‐donating group to modulate intramolecular charge transfer (ICT). In addition, the pronounced increase in near‐infrared absorption upon protonation of the indole unit also enables the probe to be used for photoacoustic imaging, thereby compensating for the limited penetration depth of fluorescence imaging. Next, to avoid protonation of the hydroxyl group under lysosomal acidic conditions, we designed the pH‐responsive probe (D‐NH_2_) by substituting the hydroxyl moiety with an amino group. To optimize the responsiveness of D‐NH_2_ to the tumor microenvironment, we systematically modified the 5‐position of its indole unit to construct a series of pH‐sensitive probes (Dx‐NH_2_). Among these derivatives, the cyano‐substituted variant CN‐D‐NH_2_ demonstrated the best protonation capability in an extracellular acidic microenvironment (A_pH = 6.5_/ A_pH = 7.5_ = 3.4, F_pH = 6.5_/ F_pH = 7.5_ = 5.9). In addition, CN‐D‐NH_2_ exhibits significant protonation differences between lysosomes and other environments (A_pH = 5_/ A_pH = 7.5_ = 8.3, F_pH = 5_/ F_pH = 7.5_ = 13.3). Subsequently, we further modified the probe based on the offensive and defensive integration (ODI) strategy proposed by our group [22]. γ‐Glutamyltransferase (GGT), which is upregulated in tumors to counteract oxidative stress, was selected as the enzymatic trigger for the defensive system, thereby enabling the development of a new generation of high‐precision tumor‐imaging probe (CN‐D‐GGT). Upon activation by GGT overexpressed on the cancer cell membrane, CN‐D‐GGT is sequestered by the abundant lysosomes within cancer cells, thereby enabling precise imaging of cancer cells (Scheme 1c).

The probe exhibited high specificity, as evidenced by its selective labeling of cancer cells in co‐culture systems (F_Tumor cell_/F_Normal cell_ = 6.8) and its ability to discriminate tumor lesions from inflammatory foci and normal tissues in vivo, thereby supporting precise tumor imaging. Notably, CN‐D‐GGT also demonstrated the ability to distinguish cancerous tissues from adjacent noncancerous tissues in clinical specimens.

## 2 Results and Discussion

### 2.1 Design and Synthesis of pH Based Activatable Probe

Metabolic reprogramming in tumor cells is often accompanied by increased lysosomal abundance, which supports the malignant phenotype by facilitating aberrant proliferation and invasion [19]. Furthermore, the acidic lysosomal lumen can protonate weakly basic molecules, thereby increasing their polarity and net positive charge and facilitating their retention within lysosomes. This feature could be leveraged to retain activated fluorophores and reduce off‐target diffusion after probe activation, yet such a strategy remains largely underexplored. Together, these properties highlight lysosomes as promising targets for the development of tumor‐imaging probes. Importantly, insufficient intracellular retention of the fluorophore may lead to its extracellular diffusion, thereby increasing background signals. Therefore, to achieve higher imaging precision, fluorescence signals should be confined, as much as possible, to the acidic tumor microenvironment.

**FIGURE 1:**
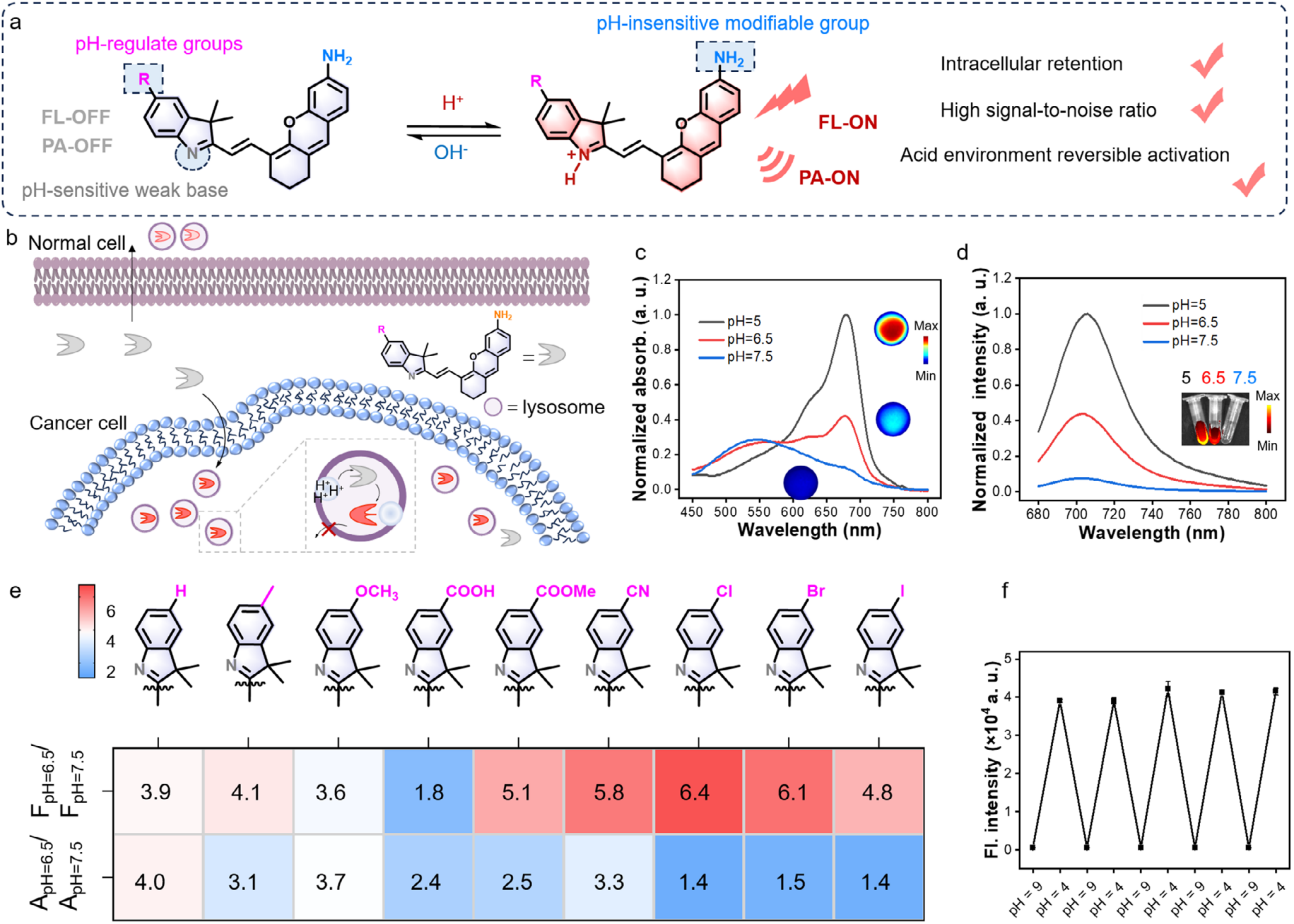
(a) Schematic of a protonation‐deprotonation responsive molecular switch for fluorescence and photoacoustic imaging. (b) Schematic of Dx‐NH_2_ exhibits brighter fluorescence in cancer cells with higher abundance and acidity of lysosomes; Dx‐NH_2_ remains silent in the normal extracellular environment, even when not captured by the lysosome of cancer cells; Dx‐NH_2_ remains for a long time after protonation, improving imaging resolution. (c) Absorption spectra and fluorescence emission spectra (d) of CN─D─NH_2_ (5 µm) in varying pH (PBS/EtOH  =  9/1, v/v, 20 mm, pH = 5, 6.5, or 7.5). (e) Relative changes in absorption and fluorescence intensity of Dx‐NH_2_ synthesized from indole substituted with 8Pearson’s correlation coefficients functional groups (methyl‐, methoxy‐, cyano‐, carboxy‐, ester‐, and halogen groups) at pH 6.5 and 7.5. (f) pH cycles of CN─D─NH_2_ (5 µm) upon addition of different acid or base treatment in buffer (PBS/EtOH  =  9/1, v/v, 20 mm). Data are mean ± SD (n = 3).

To develop molecular probes capable of precisely targeting lysosomes in cancer cells (pH 4.5–5.5), we designed and synthesized D‐NH_2_, a pH‐responsive probe. DFT calculations showed that protonation of the indole moiety under acidic conditions led to a pronounced decrease in the LUMO energy level and a reduced HOMO‐LUMO gap, accompanied by a lower S_0_→S_1_ transition energy (Figure S1). These results suggest that protonation enhances the electron‐accepting character of the indole‐containing unit and strengthens the ICT process. The probe was designed to remain nonfluorescent in the normal extracellular environment (pH 7.4) and to be activated only after internalization into lysosomes (Scheme S1). However, D‐NH_2_ exhibited little change in its protonation state over the pH of 5.0 and 6.5. In addition, its fluorescence emission at 708 nm showed only an approximately 3.8‐fold increase when the pH decreased from 7.5 to 6.5, indicating that the probe did not achieve the desired pH responsiveness (Figure S2a,b). Therefore, we next sought to tune the protonation–deprotonation equilibrium of the indole unit in D‐NH_2_, with the aim of developing probes that satisfy the above design criteria. For this purpose, a series of probes (Dx‐NH_2_) is synthesized for subsequent performance evaluation. These probes were modified at the 5‐position of the indole moiety with eight distinct substituents, namely methyl, methoxy, cyano, carboxyl, ester, fluoro, chloro, and bromo groups. Upon protonation of the indole unit, Dx‐NH_2_ exhibits enhanced ICT and increased near‐infrared absorption, thereby enabling dual‐modal imaging for high‐resolution fluorescence imaging and deep‐tissue photoacoustic imaging (Figure 1a,b).

Subsequently, the pH‐dependent fluorescence and absorption properties of the Dx‐NH_2_ series were systematically assessed to identify the optimal probe candidate. Specifically, the probe responses were evaluated under representative biological pH conditions, including those of lysosomes (pH 5.0), the tumor microenvironment (pH 6.5), and the normal extracellular environment (pH 7.5). The results showed that halogen‐substituted probes (Cl─D─NH_2_, Br─D─NH_2_, and I─D─NH_2_) exhibited the most significant difference in absorption between pH 5.0 and 6.5. In addition, these derivatives displayed considerable fluorescence contrast values of 6.28, 6.09, and 4.77, respectively, between pH 6.5 and 7.5. However, the negligible absorption changes of these probes within this pH range would result in only minimal differences in photoacoustic signals (Figure S2c–h). The absorption and emission spectra indicated that COOH─D─NH_2_ exhibited the weakest protonation behavior among the derivatives in this series (Figure S2i,j). Notably, the CH_3_O‐substituted probe showed negligible fluorescence emission even under acidic conditions, which may be due to the low quantum yield caused by the weak ICT effect in CH_3_O─D─NH_2_ (Figure S2k,l). The cyano‐, methyl‐, and ester‐functionalized probes exhibited the desired pH‐responsive behavior (Figure 1c,d; Figure S2m–p). Specifically, these derivatives showed pronounced protonation changes between pH 5.0 and 6.5, as well as between pH 6.5 and 7.5, accompanied by consistent changes in their fluorescence and absorption spectra. In summary, we systematically compared the relative fluorescence intensities and absorption changes of all pH‐responsive probes under three biologically relevant pH conditions (pH 5.0, 6.5, and 7.5) and across their pairwise comparisons (Figure 1e; Figure S3). The results showed that CN─D─NH_2_ exhibited the most pronounced protonation‐dependent differences between the lysosomal condition and the other relevant environments. Therefore, CN─D─NH_2_ was selected for subsequent investigations due to its optimal performance characteristics. Subsequently, we evaluated the protonation behavior of CN─D─NH_2_ under dynamically changing pH conditions (Figure 1f). The results showed that CN─D─NH_2_ underwent rapid and reversible protonation–deprotonation cycling in response to pH changes, confirming its favorable performance as a pH‐responsive probe.

### 2.2 CN─D─NH_2_ for Live‐Cell Imaging

**FIGURE 2:**
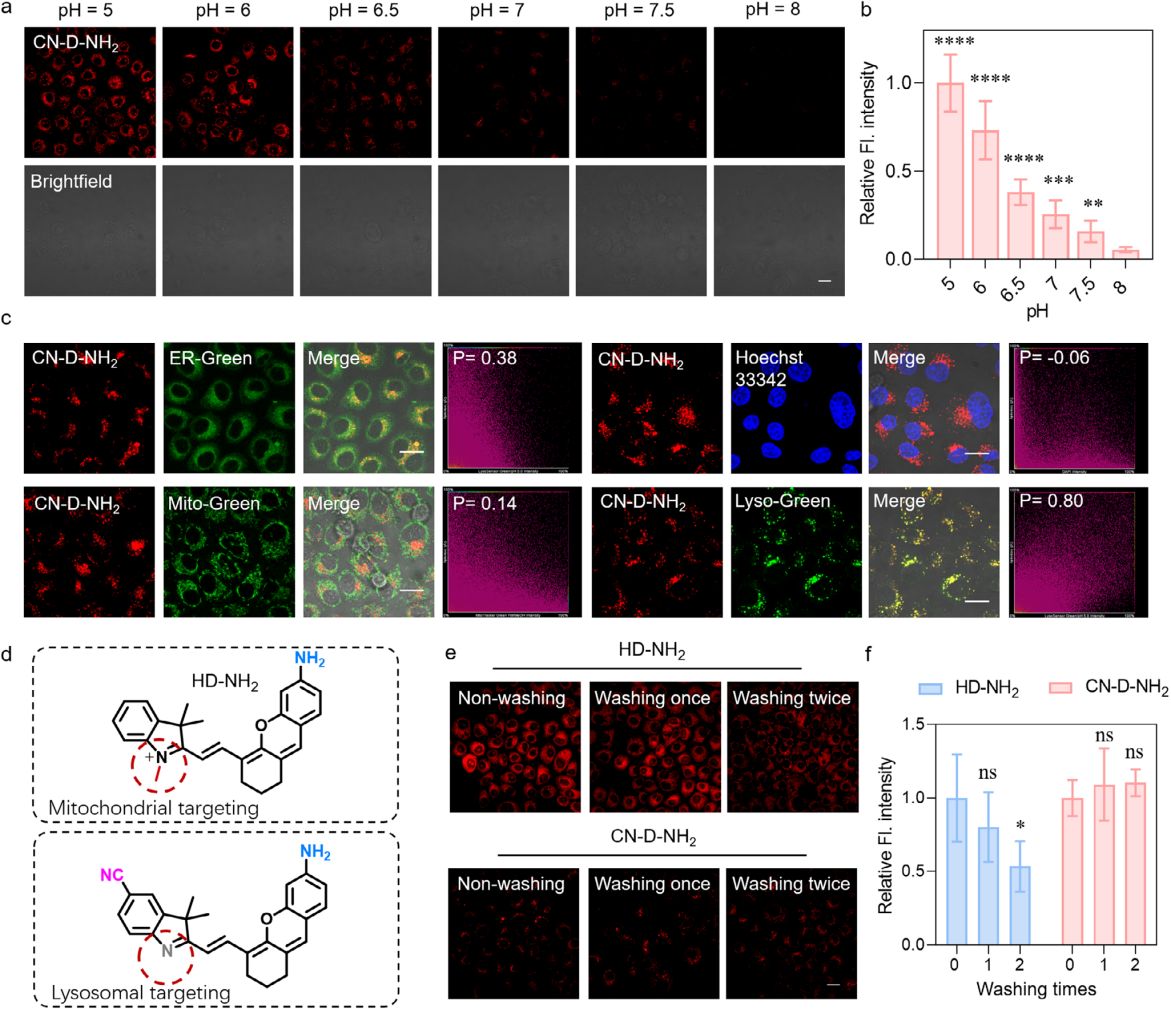
(a) Representative fluorescence images of CN─D─NH_2_ (5 µm) in different intracellular pH environments after pretreatment with nigericin (10 µg/mL) for 1 h. (b) Quantification of fluorescence intensity from panel (a). Error bars are presented as mean ± standard deviation (SD), n = 3, ^**^ *p* < 0.01, ^***^ *p* < 0.001, ^****^ *p* < 0.0001, compared with the pH 8. (c) Confocal images of live HepG2 cells treated with CN─D─NH_2_ (5 µm), co‐stained with commercial organelle trackers: ER‐Tracker Green (200 nm), Mito‐Tracker Green (200 nm), Lyso‐Tracker Green (200 nm), Hoechest 33342 (200 nm). (d) Chemical structures of HD─NH_2_ and CN─D─NH_2_. (e) Representative fluorescence images of HepG2 cells treated with HD─NH_2_ and CN─D─NH_2_, and then washed with serum‐free medium. (f) Quantification of fluorescence intensity from panel (e). Error bars are presented as mean ± standard deviation (SD), n = 5, ^*^ *p* < 0.05, “ns” for not significant, compared with non‐washing. Microscopy conditions: Mito/Lyso/ER‐Tracker Green, λ_ex_ = 488 nm, detection range 500–550 nm. CN‐D‐NH_2_, λ_ex_ = 640 nm, detection range 663–738 nm. Scale bars, 20 µm.

We next investigated the pH‐responsive behavior of CN─D─NH_2_ in live cells, after first confirming its biocompatibility (Figure S4). Then, the cells were pretreated with nigericin (10 µg/mL), a K^+^ ionophore [23]. Subsequently, cells were incubated in high‐K^+^ buffers of different pH for 1 h to establish defined intracellular pH conditions. The fluorescence images revealed that CN─D─NH_2_ exhibited a distinct pH‐dependent fluorescence response in living cells (Figure 2a,b). CN─D─NH_2_ exhibited maximum fluorescence emission under acidic conditions, whereas its fluorescence intensity decreased markedly under neutral to weakly alkaline conditions. At pH 5.0, premature protonation of CN─D─NH_2_ reduced its lysosome‐specific localization, leading to cytoplasmic fluorescence distribution (Figure 2a,b). By contrast, at pH 7.0, the observed fluorescence signal originated predominantly from the fraction retained within lysosomes. These behaviors are consistent with the protonation‐dependent activation mechanism of the probe. Subsequently, the subcellular localization of CN─D─NH_2_ was assessed by colocalization with commercial organelle trackers. Representative fluorescence images showed a high Pearson’s correlation coefficients (PCC) (> 0.8) between CN─D─NH_2_ and Lyso‐Tracker, whereas much lower PCC (< 0.4) were observed for other organelle trackers (Figure 2c). These results demonstrate that CN─D─NH_2_ exhibits excellent lysosomal localization. Moreover, CN─D─NH_2_ was rapidly internalized by living cells within 5 min (Figure S5), indicating efficient cellular uptake.

To verify the enhanced intracellular retention of lysosome‐targeted CN─D─NH_2_, living cells were incubated separately with CN─D─NH_2_ and HD─NH_2_ for 1 h (Figure 2d). After multiple washes with serum‐free medium, intracellular retention was evaluated by monitoring the decay of cellular fluorescence. HD─NH_2_ exhibited fluorescence leakage after washing twice, whereas CN─D─NH_2_ maintained constant fluorescence in cells (Figure 2e,f). These results demonstrate the superior intracellular retention capacity of CN─D─NH_2_ in living cells.

### 2.3 Design and Synthesis of a Novel High‐Precision Probe Based on ODI Strategy

**FIGURE 3:**
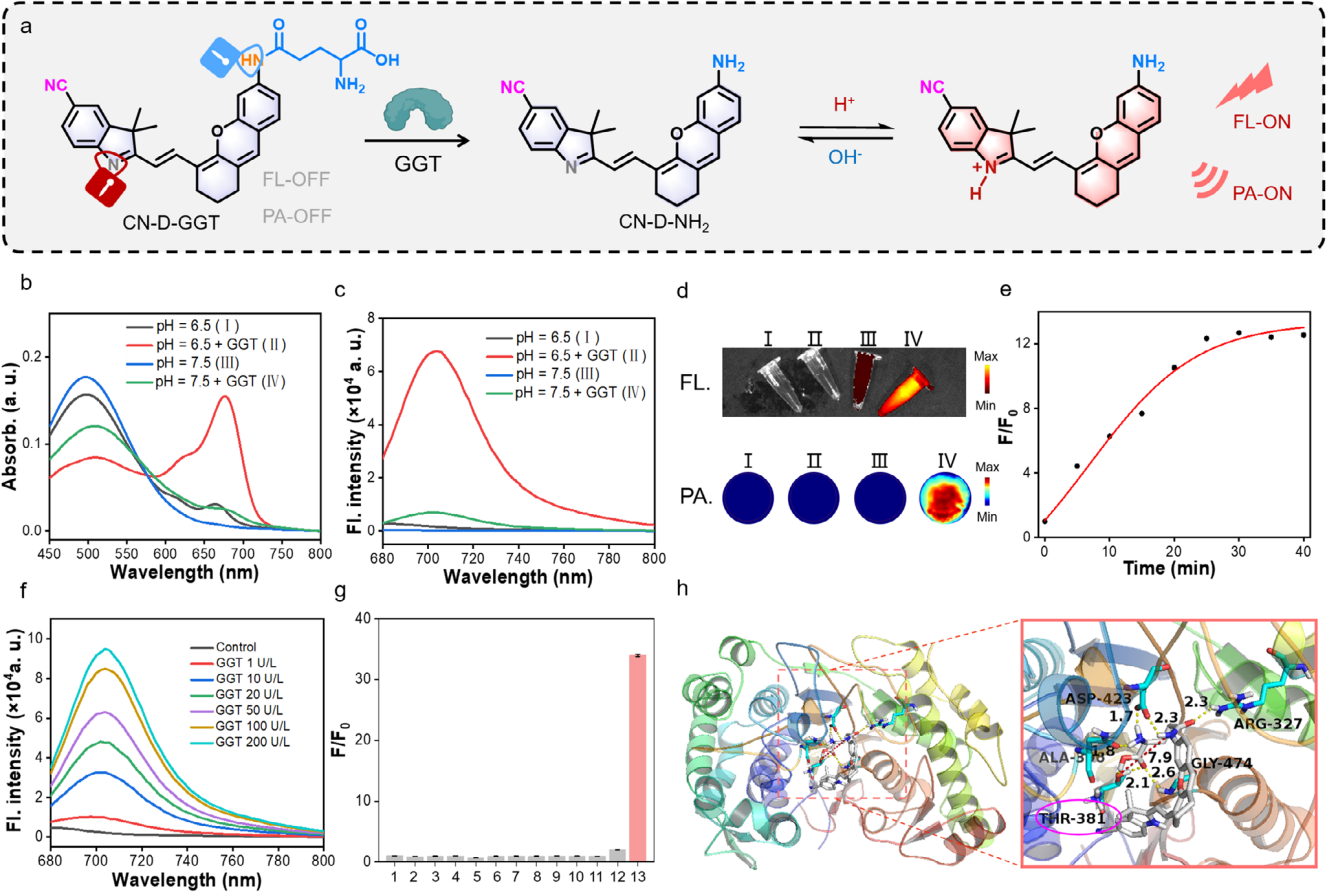
(a) Schematic of a protonation‐deprotonation and GGT responsive probe (CN─D─GGT) switch for fluorescence and photoacoustic imaging. The absorption (b) and fluorescence emission spectra (c) of CN─D─GGT (5 µm) either without or with GGT (100 U/L), and acidic environment (pH = 6.5). (d) Fluorescence and photoacoustic images of CN─D─GGT under four different solution conditions. (I) PBS at pH 6.5; (II) PBS at pH 6.5 containing GGT; (III) PBS at pH 7.5 containing GGT; (IV) PBS at pH 7.5. (e) Time‐dependent fluorescence response (708 nm) of CN‐D‐GGT (5 µm) with GGT (100 U/L) at pH 6.5. (f) Fluorescence emission spectra of CN─D─GGT (5 µm) incubated with GGT (0–200 U/L) at pH 6.5. (g) Fluorescence enhancement of CN─D─GGT, at 708 nm in the presence or absence of various analytes, 1: Blank, 2: HClO (100 µm), 3: H_2_O_2_ (100 µm), 4: Vitamin C (500 µm), 5: NaHS (100 µm), 6: GSH (1 mm), 7: Esterase (0.5 U/L), 8: ALP (100 U/L), 9: β‐Gal (200 U/L), 10: APN (100 ng/mL), 11: LAP (100 U/L), 12: pH = 6.5, 13: GGT (100 U/L) at pH = 6.5. (h) Molecular docking results of CN─D─GGT [H+] and GGT (PDB: 4ZBK). The yellow line represents a hydrogen bond; The red line represents the distance between the active center and the cutting position.

Encouraged by these findings, we further functionalized CN─D─NH_2_ according to our previously reported ODI strategy [22] to develop a new generation of high‐precision imaging probes. The increased abundance of lysosomes, along with elevated acidity in the tumor microenvironment, collectively promote tumor‑aggressive behaviors (such as cancer cell metabolic reprogramming, basement membrane degradation, and macrophage polarization) that drive rapid proliferation, constituting an “attack system” of tumors [24, 25, 26]. Therefore, we employed GGT, a membrane‐associated enzyme that enhances GSH synthesis and neutralizes reactive oxygen species in cancer cells, as a substrate‐responsive element of the tumor “defense system” [27, 28, 29]. Following the above concept, a dual lock probe, CN─D─GGT, that is activated by both acidic environment and GGT, was successfully synthesized (Figure 3a). First, CN─D─GGT achieves initial selectivity through specific activation by GGT on the surface of cancer cells within the tumor microenvironment. Subsequently, the elevated lysosomal abundance in cancer cells, coupled with acid‐triggered protonation and intracellular retention, enables spatiotemporal confinement and amplification of the imaging signal. This mechanism endows CN─D─GGT with the potential for high‐precision tumor imaging. Synergistic quenching of the fluorophore by the two recognition motifs minimizes background fluorescence, whereas fluorescence is restored only in the simultaneous presence of an acidic environment and GGT, thereby enabling high‐contrast and precise tumor imaging.

To evaluate its performance, CN─D─GGT was incubated under four different conditions, followed by spectroscopic analysis. Spectroscopic evaluation was conducted under four conditions: (I) PBS at pH 6.5, chosen to simulate the tumor microenvironment in which CN─D─GGT is activated by membrane‐associated GGT; (II) PBS at pH 6.5 containing GGT; (III) PBS at pH 7.5 containing GGT; and (IV) PBS at pH 7.5. Absorption and fluorescence emission spectra revealed that CN─D─GGT showed an absorption maximum at 512 nm with negligible fluorescence in PBS at pH 7.5. As the pH was lowered to 6.5, a pronounced redshift (680 nm) in the absorption spectrum was observed, while the fluorescence intensity remained essentially unchanged. Although the probe could be activated by GGT in PBS (pH = 7.5), the ICT effect of CN─D─GGT is continuously suppressed by the low protonation tendency of the indole moiety under neutral to alkaline conditions, which explains the merely modest rise in fluorescence and the slight redshift in absorption. As expected, a pronounced fluorescence enhancement (∼32‐fold) together with a noticeable wavelength shift in absorption was observed for CN─D─GGT in PBS at pH 6.5 containing GGT (Figure 3b,c). In addition, these solutions exhibited significant intergroup signal differences in both fluorescence and photoacoustic imaging systems (Figure 3d). These results demonstrate that CN─D─GGT exhibits dual‐biomarker‐dependent fluorescence/photoacoustic activation and, together with its advantages of extremely low background and rapid response, holds considerable promise for practical applications.

Subsequently, we investigated the activation kinetics of CN─D─GGT in the presence of GGT at pH 6.5. As shown in the Figure 3e, the fluorescence reaches the plateau within 30 min, indicating the rapid response kinetics of CN─D─GGT. Subsequently, the concentration‐dependent activation of CN─D─GGT to GGT was evaluated in PBS at pH 6.5. The fluorescence, absorption, and photoacoustic signals all increased with increasing GGT concentration and approached saturation at approximately 100 U/L (Figure 3f; Figure S6a,b). These results demonstrate that CN─D─GGT exhibits a robust concentration‐dependent response to GGT under mildly acidic conditions. Then, an in vitro selectivity assay was performed to assess the specificity of CN─D─GGT in complex biological environments (Figure 3g). The results demonstrated that CN─D─GGT retained selective activation under complex conditions, supporting its potential for specific tumor imaging. Moreover, molecular docking revealed that CN─D─GGT can bind to the active pocket of GGT (PDB: 4ZBK) [30], with binding energies of −6.96 kcal/mol (Figure S7). Notably, protonated CN─D─GGT ([H+] form) exhibited a lower binding energy (−8.79 kcal/mol) and a shorter distance to the catalytic residue Thr381 in the GGT active site (Figure 3h). These results suggest that protonation facilitates the interaction of CN─D─GGT with GGT, thereby promoting more efficient activation under the mildly acidic extracellular conditions of cancer cells.

### 2.4 CN─D─GGT for Live‐Cell Imaging

**FIGURE 4:**
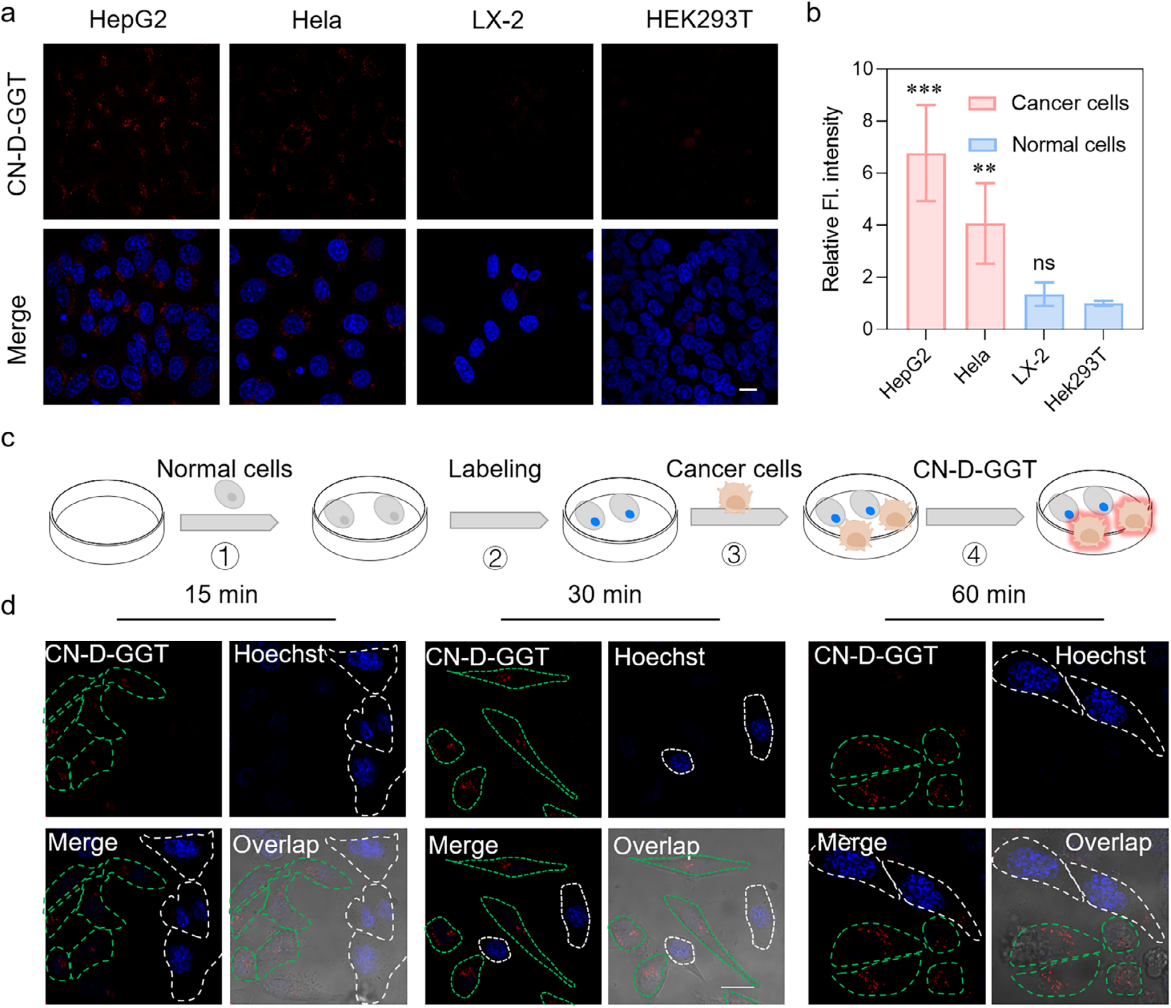
(a) Representative fluorescence images of cancer cells (HepG2 and Hela) and normal cells (LX‐2, and HEK293T) after 1 h of incubation with CN─D─GGT (5 µm). (b) Quantification of fluorescence intensity from panel (a). (c) Preparation for imaging the cell co‑culture system by CN─D─GGT. (d) Representative fluorescence images of the co‐culture system after incubation with CN‐D‐GGT for different time periods. The green enclosed area represents cancer cells, while the white enclosed area represents normal cells. Error bars are presented as mean ± standard deviation (SD), n = 5. Statistical significance is indicated by ^**^ *p* < 0.01, ^***^ *p* < 0.001. ns, not significant (*t*‐test).

Based on its potential to discriminate between normal and cancer cells, CN─D─GGT was incubated with cancer cells (including HepG2 cells and Hela cells) or normal cells (including LX‐2 cells and HEK293T cells) for 1 h. Prior to this, the biocompatibility of CN─D─GGT has been confirmed (Figure S8). Fluorescence images in all cell lines were subsequently acquired by confocal microscopy (Figure 4a). Representative fluorescence images showed an approximately 6‐fold higher fluorescence of CN─D─GGT in HepG2 cells than in normal cells. A similar trend was also observed in photoacoustic images (Figure S9). This enhanced signal is likely attributable to the combined effects of membrane GGT overexpression and the increased abundance of lysosomes in HepG2 cells (Figure 4b). The intracellular distribution of CN─D─GGT shows high colocalization with Lyso‐Tracker, indicates that CN─D─GGT is further captured by lysosomes after being activated by GGT (Figure S10). Furthermore, both the fluorescence and photoacoustic signals of CN─D─GGT in HepG2 cells can be suppressed by DON, a GGT inhibitor [31], confirming that CN‐D‐GGT activation is dependent on GGT activity (Figure S11).

Encouraged by the results, we next established a cell co‐culture system to better simulate the complex cellular composition of the tumor microenvironment. To establish the cell co‐culture model, normal cells were first prelabeled with Hoechst 33342. HepG2 cells were then seeded into the same confocal dish and co‐cultured with the normal cells for 24 h (Figure 4c). Before imaging, the co‐culture system was incubated with CN─D─GGT for 15, 30, or 60 min. Confocal images showed that the normal cells, identified by Hoechst nuclear staining, exhibited negligible fluorescence, whereas the unlabeled HepG2 cells displayed distinct fluorescence signals (Figure 4d). These results indicate that CN─D─GGT can effectively discriminate cancer cells from normal cells even under co‐culture conditions, highlighting its potential for precise tumor imaging.

### 2.5 CN─D─GGT for In Vivo and Tissue Imaging

However, the aforementioned experiments mainly focused on monitoring intracellular fluorescence. Therefore, we further sought to evaluate the application potential of CN‐D‐GGT in the complex extracellular tumor microenvironment. To further evaluate its performance in a more physiologically relevant setting, CN─D─GGT was injected separately into the tumor and muscle tissues of tumor‐bearing mice. After 10 min, the injected tissues were harvested, sectioned, and subjected to fluorescence imaging (Figure S12). Notably, strong fluorescence was observed only in tumor tissues, whereas muscle tissues showed minimal signal, indicating selective activation of CN─D─GGT in the tumor and its potential to distinguish tumor tissues from normal tissues.

In addition to normal tissues, inflammatory lesions can also interfere with the accurate diagnosis of cancer [32]. The substantial similarity in biomarker expression between tumors and inflammatory lesions, particularly in the context of inflammation‐associated tumorigenesis, makes their accurate differentiation clinically challenging [33]. Currently, few fluorescent probes can reliably discriminate cancerous tissues from both normal and inflamed tissues. Encouraged by the satisfactory performance of CN─D─GGT, we next evaluated its ability to address this challenge.

**FIGURE 5:**
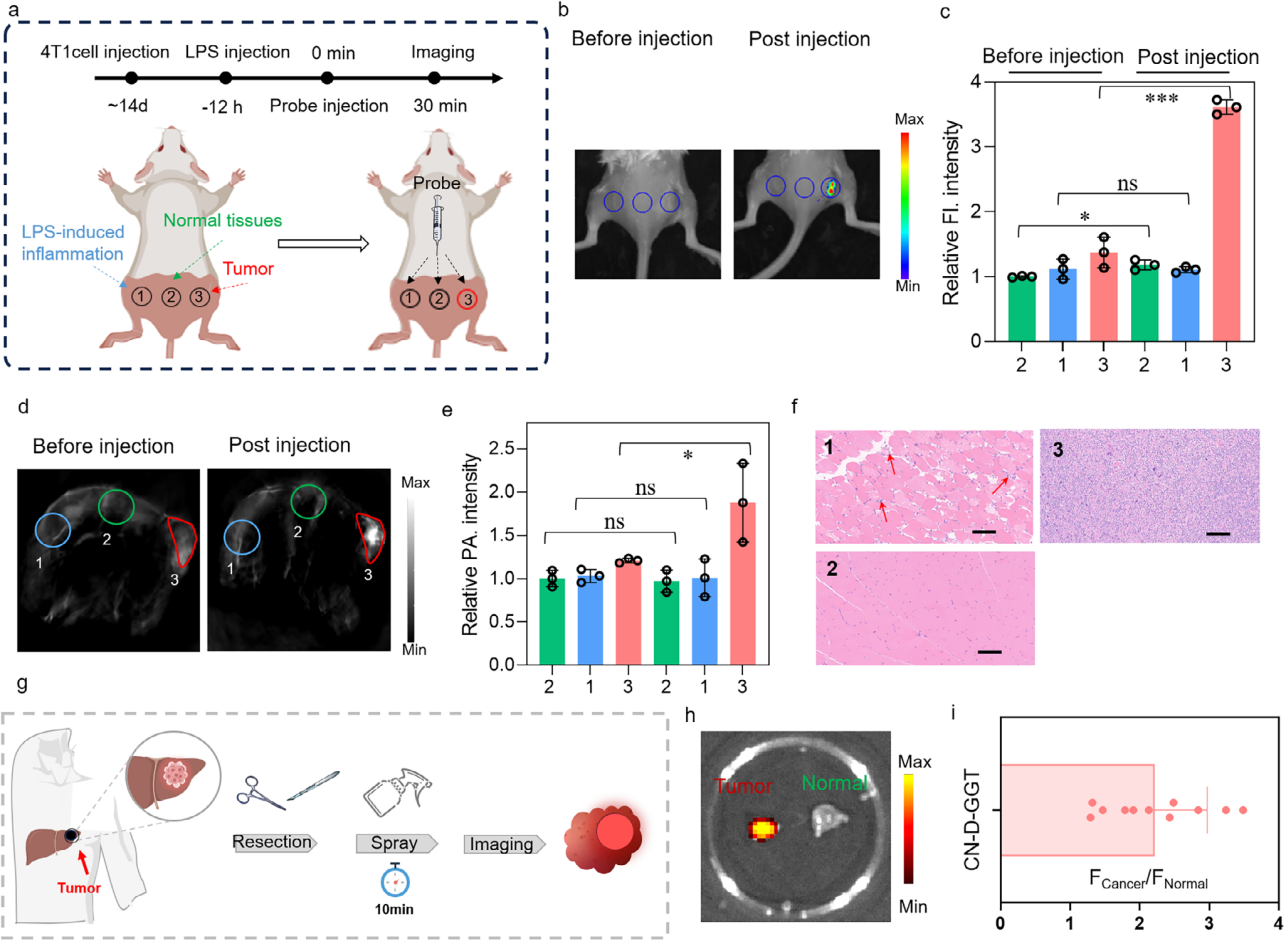
(a) Schematic preparation of three tissue models for CN─D─GGT imaging, including normal, tumor, and inflamed tissues. (b) Representative fluorescence images of three tissue models before and after in situ injection of CN‐D‐GGT (50 µm, 25 µL). (c) Quantification of fluorescence intensity from panel (b). Error bars are presented as mean ± standard deviation (SD), n = 3. ^*^ *p* < 0.05, ^***^ *p* < 0.001, ns, not significant (t‐test). (d) Representative photoacoustic images of three tissue models before and after in situ injection of CN‐D‐GGT (50 µm, 25 µL). (e) Quantification of fluorescence intensity from panel (d). Error bars are presented as mean ± standard deviation (SD), n = 3. ^*^ *p* < 0.05, ns, not significant (*t*‐test). (f) The H&E staining of three tissue models in (b,d). Scale bar: 50 µm. (g) Illustration of HCC clinical sample processing and fluorescence imaging. (h) Fluorescence images of tumor and adjacent tissues after incubation with CN─D─GGT (50 µM) for 10 min. (i) Quantification of fluorescence intensity from panel (h). Error bars are presented as mean ± standard deviation (SD), n = 11.

For this purpose, an acute inflammation model was established by subcutaneous injection of lipopolysaccharide (LPS) into the thigh of tumor‐bearing mice for 12 h (Figure 5a). Subsequently, CN─D─GGT was locally injected into normal tissue, inflamed tissue, and tumor tissue, followed by imaging analysis 30 min later. Representative fluorescence images showed that observable signals were detected only at the tumor site, whereas normal and inflamed tissues exhibited negligible signal changes (Figure 5b,c; Figure S13). Consistently, representative photoacoustic images showed the same trend (Figure 5d,e; Figure S13). Subsequent pathological analysis of the probe‐injected regions confirmed the successful establishment of the three models: tumor, inflammation, and normal (Figure 5f). Moreover, CN─D─GGT exhibited good biocompatibility and did not cause noticeable tissue damage (Figure S14). These results suggest that CN─D─GGT can effectively distinguish tumor tissues from both normal and inflamed tissues, supporting its potential for precise tumor imaging in complex pathological environments. Subsequently, the tumors were pretreated with an inhibitor (DON) before intratumoral injection of CN─D─GGT. Representative fluorescence and photoacoustic images showed that the signal enhancement was effectively suppressed after inhibitor treatment (Figure S15). These findings further suggest that probe activation is closely linked to the GGT in vivo, thus providing additional evidence for its imaging specificity.

Encouraged by the above results, we further applied CN─D─GGT to the diagnosis of human hepatocellular carcinoma (HCC) tissues and evaluated its feasibility for distinguishing HCC from adjacent tissues (Figure 5g). Clinical HCC specimens were incubated with CN─D─GGT (50 µm) prior to fluorescence imaging (Figure 5h,i; Figure S16). Representative fluorescence images indicate that CN─D─GGT produced the highest tumor‐to‐normal (T/N) ratio (2.22‐fold) after 10 min of incubation. These results demonstrate that CN─D─GGT can effectively differentiate HCC tissue from adjacent tissue in clinical specimens, highlighting its potential for rapid clinical diagnosis.

## 3 Conclusion

In summary, we developed a series of lysosome‐targeted activatable probes (Dx‐NH_2_) by leveraging tumor‐associated extracellular acidosis and increased lysosomal abundance, based on a modified hemicyanine scaffold. Compared with previously reported pH‐responsive fluorescent probes, the Dx‐NH_2_ offers more readily tunable protonation behavior, along with favorable lysosomal targeting and retention. In addition, Dx‐NH_2_ contains a functionalize site that enables the introduction of other responsive moieties for the construction of activatable probes with low background and high signal‐to‐background ratios. Then, systematic screening identified CN─D─NH_2_ as the optimal reversible pH‐responsive probe, and further implementation of our ODI strategy with GGT as the enzymatic trigger afforded the dual‐site activatable probe CN─D─GGT. Owing to its extremely low background, stringent dual‐feature activation, and lysosomal retention‐enhanced signal confinement, CN─D─GGT effectively reduced both false‐positive and false‐negative interference, enabling the potential for high‐precision tumor imaging. Notably, CN─D─GGT distinguished cancer cells from normal cells with an approximately 6‐fold signal difference and discriminated tumors from both normal and inflamed tissues with T/N or T/I signal ratios greater than 3.5. It further demonstrated the ability to differentiate tumor from adjacent tissue in clinical HCC specimens, underscoring its promise for translational tumor detection and rapid clinical tissue diagnosis. These results demonstrate the potential of the probe for precise imaging applications. In surgical resection, the probe may provide real‐time visualization of lesion tissues and their boundaries through rapid and accurate imaging. Such performance could assist surgical decision‐making, reduce the likelihood of lesion omission, and minimize excessive resection of normal tissues. Overall, these results highlight the promising translational potential of the probe.

## Conflicts of Interest

The authors declare no conflicts of interest.

## Supporting information




**Supporting File**: advs75664‐sup‐0001‐SuppMat.docx.

## Data Availability

The data that supports the findings of this study are available in the supplementary material of this article
