# Supplementary material for: Synergistic Probe Combining Lysosome Anchoring and Tumor Defense System Targeting for Precise Tumor Visualization
Source: Adv Sci (Weinh). 2026 May 13;13(44):e75664. doi: 10.1002/advs.75664 (PMC13335931; doi:10.1002/advs.75664)
Supplement: Supplementary file 1 [file ADVS-13-e75664-s001.docx]

**Supporting Information**

**Synergistic Probe Combining Lysosome Anchoring and Tumor Defense System Targeting for Precise Tumor Visualization**

Yang Shen,^[a,b]^ Jiawei Li,^[b]^ Ruixi Yi,^[b]^ Ling Shi,^[b]^ Wei Li,^[b,c]^ Sulai Liu,^[a],^* Lin Yuan^[b]^

[a]	Dr. Y. Shen, Prof. Dr. S. Liu.

Department of Hepatobiliary Surgery, Central Laboratory,

Hunan Provincial People's Hospital (The First Affiliated Hospital of Hunan Normal University)

Changsha, 410005, (PR China)

E-mail: liusulai@hunnu.edu.cn

[b]	Dr. Y. Shen, J. Li, R. Yi, Dr. L. Shi, Dr. W. Li, Prof. Dr. L. Yuan
State Key Laboratory of Chemo and Biosensing, College of Chemistry and Chemical Engineering
Hunan University
Changsha 410082 (PR China)

[c]	Dr. W. Li
School of Chemistry and Molecular Engineering, Nanjing Tech University, Nanjing 211816 (PR China)

**Table of Contents**

[Methods	S-2](#_Toc159355066)

[Synthesis Procedure	S-5](#_Toc159355067)

[Supplementary Figures	S-10](#_Toc159355068)

[NMR and Mass Spectra	S-18](#_Toc159355069)

[References	S-31](#_Toc159355070)

**Methods**

Detailed synthesis procedures and spectroscopic data for all compounds are available in the Supplementary Information.

**Materials and general methods**

All chemical reagents and solvents were obtained commercially from Bide Pharmaceutical Technology, Energy Chemical, and Shanghai Titan Technology Company Limited, and were used without further purification. Reaction progress was monitored by thin-layer chromatography (TLC) on silica gel plates, and products were purified by column chromatography using silica gel (100–200 mesh) from Shanghai Haohong Biomedical Technology Company Limited. γ-Glutamyl transferase (GGT) were acquired from Shanghai Yuanye Biotechnology Company Limited. ¹H NMR and ¹³C NMR spectra were acquired on a Bruker-400 spectrometer using tetramethylsilane (TMS) as an internal standard. Mass spectra were performed using an LCQ Advantage ion trap mass spectrometer (Thermo Finnigan) and matrix-assisted layer desorption/ionizationtime-of-flight mass spectrometry (MALDI-TOF/MS, UltrafleXtreme, Bruker). High-resolution electron spray mass spectra (HRMS) were obtained from Orbitrap Exploris 120 (Analysis and Testing Center in Hunan University). Absorption and fluorescence spectroscopic studies were performed in a UV-1800 ultraviolet and visible spectrophotometer (Shimadzu Corporation, Japan) and an Edinburgh spectrofluorometer FS5 (Edinburgh Innovations, Germany). Cell imaging was performed on Nikon A1 plus confocal microscope (Nikon, Japan). In vivo imaging was carried out on an IVIS Lumina XR (IS1241N6071) imaging system. All photoacoustic images were analyzed and collected at various time points by an InVision 256-TF imaging system (iTheraMedical).

**Measurements of UV-Vis and fluorescence spectroscopy**

Stock solutions of Dx-NH_2_ were prepared in DMSO at 1 mM. Typically, DMSO is used as the solvent to prepare stock solutions of Dx-NH_2_ at 10 mM, which are stored at −20 °C. Upon receipt, GGT is immediately dissolved in PBS to form an aqueous solution of 100 U/mL for routine experiments. To preserve enzymatic activity, all these solutions are stored at −80 °C until use. For spectral measurements, 1 µL of dye or probe stock solution (10 mM) was added to 1 mL of an aqueous solution—either with varying pH (PBS/EtOH = 9/1, v/v, 20 mM, pH 5, 6.5, or 7) and incubated at 30 °C for 30 min. The reaction mixture was then transferred to a quartz cuvette for absorbance or fluorescence measurement. For fluorescence measurement, the excitation wavelength is set to 660 nm.

**Molecular docking of protein targets**

The compound ligand for this docking, ref was constructed using ChemDraw software and imported into Chem3D software using MM2 module for optimisation as well as energy minimisation and saved as a sdf format file as the ligand molecule for molecular docking. Afterwards, it was imported into Maestro 12.8 software and optimised using LigPrep module and the force field was selected as OPLS3e.CA (PDB ID: 4ZBK) protein structure was obtained from RCSB database. Protein structures were processed in Maestro 11.9 to remove any unreasonable structures, and Schrodinger's Protein Preparation Wizard was used to process the proteins for energy minimisation and geometry optimisation. Molecular docking was processed and optimised by the Glide module in the Schrödinger Maestro software. Protein processing utilised the Protein Preparation Wizard module. Receptors were pre-processed, optimised and minimised (constrained minimisation using the OPLS3e force field). Compound structures were prepared according to the default settings of the LigPrep module. For screening in the Glide module, the prepared receptors were imported and their binding sites were identified based on the protein active site residues (THR381, ASP423, GLY474, ARG327) and the box size was set to 10Åx10Åx10Å. Finally, molecular docking and screening were performed by Induced Fit docking (IFD) method.

**Confocal live cell imaging**

In this study, all cells (HepG2, LX-2, HEK293T, Hela, L929) were cultured in DMEM supplemented with 10% fetal bovine serum (FBS) and maintained in a humidified incubator at 37 °C with 5% CO_2_.

**Cell cytotoxicity**

HepG2 cells were seeded in 96-well plates at a density of 1 × 10^4^ cells per well and cultured for 48 h. Subsequently, the cells were treated with varying concentrations of probes and incubated for 24 h. After treatment, MTT reagent (0.5 mg/mL) was added to each well and the plates were incubated at 37 °C for 4 h. The resulting formazan violet crystals were dissolved in DMSO (200 µL per well) with additional shaking at 37 °C for 30 min. Absorbance was measured at 480 nm using a multimode microplate reader. Cell viability was calculated as follows: (mean absorbance of treated group / mean absorbance of control group) × 100%.

**Co localization experiment**

2–4 × 10⁴ HepG2 cells were seeded into confocal dishes and cultured in a sterile incubator at 37 °C with 5% CO₂ for 36 h. The dishes were then washed twice with warm DPBS to remove cellular debris or metabolites, followed by incubation with DMEM pre‑mixed with D-CN-NH_2_ (5 µM). 30 min later, commercially available subcellular localization reagents (200 nM each: MitoTracker Green, ER‑Tracker Green, Lyso-Tracker Green, and Hoechst 33342) were added and co‑incubated with the cells for 15 min. Prior to imaging, the cells were washed with warmed serum‑free medium to remove excess dye that had not entered the cells. Microscope settings: excitation wavelength λ_ex_ = 640 nm; detection range 663–738 nm.

**Imaging of different pH levels within living cells**

First, high‑potassium buffers with pH values of 5, 6, 6.5, 7, 7.5 and 8 were prepared using a pH meter. HepG2 cells that had been seeded in confocal dishes for 36 h were incubated with nigericin for 1 h. After removal of the culture medium, the cells were treated with the different pH‑adjusted high‑potassium buffers to alter the intracellular pH. 30 min later, the dye was added and co‑incubated with the cells for 30 min. No washing step was performed prior to imaging. Microscope settings: excitation wavelength λ_ex_ = 640 nm; detection range 663–738 nm.

**Imaging of mixed culture system of cancer cells and normal cells**

L929 cells (~1×10⁴) were seeded in confocal dishes on day 1 and maintained in 10% FBS‑containing medium for 24 h. The following day, the cells were stained with 200 nM Hoechst 33342 for 3 h and then rinsed with warm serum‑free medium to eliminate unbound Hoechst 33342. Subsequently, approximately 1×10⁴ fast‑growing HepG2 cells were seeded onto the pre‑stained L929 cells and co‑cultured for 24 h. On the third day, cells were rinsed twice with warm DPBS to clear dead cells and metabolites, followed by incubation for 30 min in DMEM containing 5 μM of CN-D-GGT. The cells were imaged directly without a subsequent wash step.

**Animal model**

All animal experiments were conducted in accordance with the Hunan Provincial Regulations on the Management of Laboratory Animals (License No.: SYXK (Xiang) 2023-0010). Female BALB/c mice (approximately 5 weeks old, weighing 16–18 g) were obtained from Hunan Slack Jingda Laboratory Animal Co., Ltd. The mice were maintained in a pathogen‑free environment and housed in sterile cages within individually ventilated cabinets. To establish a mouse model bearing 4T1 cells, 1×10⁷ 4T1 cells suspended in 50 μL of serum‑free medium were injected subcutaneously into the right flank of each mouse. The tumor diameter reached approximately 3 mm. Normal mice used in the experiment were those that received no treatment. Acute subcutaneous inflammation was induced in tumor‑bearing mice via a single LPS injection (300 mg/kg) into the thigh, followed by a 12 h incubation period.

**In vivo imaging**

Prior to in vivo imaging, mice were anesthetized by inhalation of 5% isoflurane in 100% oxygen. For both subcutaneous and intratumoral injections, CN-D-GGT (50 μM) was prepared in ultrapure water without buffering capacity. Imaging was performed on female BALB/c mice using a Caliper VIS Lumina XR small‑animal optical in vivo imaging system. Imaging parameters: excitation wavelength λ_ex_ = 675 nm; emission wavelength λ_em_ = 695–770 nm.

**Photoacoustic imaging of solutions, cells, and living animals**

The probe was first introduced into solutions representing various test environments. Each mixture was then transferred to a 300 μL microcentrifuge tube, and photoacoustic images were collected at 680 nm on a photoacoustic imaging system (iTheraMedical). For the preparation of cell imaging samples: After co-incubating the cells with CN-D-GGT for 30 minutes, residual culture medium was removed by washing with DPBS. Following trypsinization, the cells were collected by centrifugation, and the resulting cell pellet was resuspended in 300 μL of DPBS for subsequent photoacoustic imaging. Prior to in vivo imaging, mice were anesthetized by inhalation of 5% isoflurane in 100% oxygen. For both subcutaneous and intratumoral injections, CN-D-GGT (50 μM) was prepared in ultrapure water without buffering capacity. Imaging was performed on female BALB/c mice using a photoacoustic imaging system (iTheraMedical). The collection wavelength of photoacoustic images is 680 nm.

**Imaging of clinical samples.**

The study was approved by the Medical Ethics Committee of Hunan Provincial People’s Hospital (The First Affiliated Hospital of Hunan Normal University) (Number: 2023–151), and all clinical samples were used in accordance with institutional guidelines and the Declaration of Helsinki after obtaining signed informed consent from all participants. Perform fluorescence imaging of tumor tissues from clinically diagnosed hepatocellular carcinoma patients. The tumor samples (about 0.5×0.2×0.2 cm) and adjacent tissues were placed into same clean dishes, which were full of CN-D-GGT (50 μM**)**, depending on the patient's source. After standing for 10 min, the imaging was performed.

**Synthesis** **Procedure**

Scheme S1. Synthetic route of Dx-NH_2_

Me-indole, Cl-indole, Br-indole, I-indole, and H-indole were purchased directly from commercial suppliers and used without further purification; MeO-indole, COOH-indole, CN-indole, and compound 1 were synthesized according to literature procedures^1-2^.

**Synthesis of D-NH₂:** A mixture of H-indole (200 mg, 1.25 mmol), compound **1** (161 mg, 0.628 mmol), and potassium acetate (122.5 mg, 1.25 mmol) was placed in a 100 mL round-bottom flask. Subsequently, acetic acid (5 mL) and acetic anhydride (5 mL) were added. The reaction mixture was then stirred at 80 °C. After 6 hours, the reaction mixture was poured into ice water, and the pH was adjusted to neutral with sodium bicarbonate. The resulting mixture was then filtered. The collected solid was dissolved and purified by column chromatography using dichloromethane (DCM) as the eluent, affording D-NO₂ as a tan solid (121 mg, yield 48.2%). D-NO₂ (100 mg, 0.25 mmol) was dissolved in methanol (20 mL). To this solution, SnCl₂·2H₂O (562.5 mg, 2.5 mmol) dissolved in concentrated hydrochloric acid (2 mL) was added dropwise under ice bath. The reaction mixture was then heated to 85 °C. After 2 hours, the reaction mixture was adjusted to neutral pH and then extracted multiple times with DCM. The combined organic extracts were dried over anhydrous sodium sulfate. After removal of the solvent under reduced pressure, the residue was purified by column chromatography to afford D-NH₂ as a tan solid (28.6 mg, yield 31.0%). ^1^H NMR (400 MHz, Chloroform-*d*) δ 8.25 (d, *J* = 16.0 Hz, 1H), 7.59 (d, *J* = 7.6 Hz, 1H), 7.29 (dt, *J* = 10.9, 7.8 Hz, 2H), 7.17 (t, *J* = 7.4 Hz, 1H), 6.83 (d, *J* = 8.1 Hz, 1H), 6.46 (s, 1H), 6.36 (s, 1H), 6.31 (d, *J* = 6.2 Hz, 2H), 2.50 (q, *J* = 6.2 Hz, 4H), 1.79 (p, *J* = 6.1 Hz, 2H), 1.46 (s, 6H). ^13^C NMR (101 MHz, Chloroform-*d*) δ 154.4, 154.0, 151.6, 148.2, 146.3, 134.0, 127.7, 126.9, 126.1, 124.6, 123.5, 121.0, 119.6, 114.2, 113.5, 110.2, 110.1, 101.2, 52.2, 29.8, 24.7, 24.5, 21.1. HRMS, m/z: [M+H] + calc. for C_25_H_25_N_2_O 369.1961, found 369.1971.

**Synthesis of Me-D-NH₂:** A mixture of Me-indole (169 mg, 0.976 mmol), compound **1**(100 mg, 0.39 mmol), and potassium acetate (95.6 mg, 0.976 mmol) was placed in a 100 mL round-bottom flask. Acetic acid (5 mL) and acetic anhydride (5 mL) were then added. The reaction mixture was stirred and heated at 80 °C overnight. On the following day, the reaction mixture was poured into ice water and neutralized with sodium bicarbonate. The resulting precipitate was collected by filtration and dissolved. The crude product was purified by column chromatography using DCM as the eluent, yielding Me-D-NO₂ as a tan solid (89 mg, 55.4% yield). Me-D-NO₂ (89 mg, 0.216 mmol) was dissolved in methanol (10 mL). To this solution, cooled in ice bath, a solution of SnCl₂·2H₂O (486 mg, 2.16 mmol) in hydrochloric acid (2 mL) was added dropwise. The reaction mixture was then heated to 85 °C and stirred under reflux for 8 hours. After 8 hours, the mixture was adjusted to neutral pH and extracted multiple times with DCM. The combined organic extracts were dried over anhydrous sodium sulfate. The solvent was removed under reduced pressure, and the residue was purified by column chromatography to afford Me-D-NH₂ as a tan solid (25.8 mg, yield 31.2%). ^1^H NMR (400 MHz, Methanol-*d_4_*) δ 8.24 (d, 1H), 7.32 (d, *J* = 7.7 Hz, 1H), 7.19 (s, 1H), 7.11 (d, *J* = 7.9 Hz, 1H), 6.88 (d, *J* = 8.1 Hz, 1H), 6.44 (s, 2H), 6.38 (d, *J* = 7.2 Hz, 1H), 6.28 (s, 1H), 2.52 (s, 4H), 2.39 (s, 3H), 1.81 (p, *J* = 6.1 Hz, 2H), 1.44 (s, 6H). ^13^C NMR (101 MHz, Chloroform-*d*) δ 167.3, 158.6, 155.2, 151.2, 143.3, 142.1, 141.2, 135.0, 131.10, 129.0, 127.8, 124.3, 122.4, 115.6, 113.3, 112.3, 111.7, 100.8, 50.7, 29.3, 25.6, 24.4, 21.5, 20.8. HRMS, m/z: [M+H] + calc. for C_26_H_27_N_2_O 383.2188, found 383.2128.

**Synthesis of MeO-D-NH₂:** A mixture of MeO-indole (147.4 mg, 0.976 mmol), compound **1** (100 mg, 0.39 mmol), and potassium acetate (76.4 mg, 0.78 mmol) was placed in a 100 mL round-bottom flask. Acetic acid (5 mL) and acetic anhydride (5 mL) were then added. The reaction mixture was stirred and heated at 80 °C overnight. The following day, the mixture was poured into ice water and neutralized with sodium bicarbonate. The resulting precipitate was collected by filtration and dissolved. The crude material was purified by column chromatography using DCM as the eluent, affording MeO-D-NO₂ as a tan solid (67 mg, 40.1% yield). MeO-D-NO₂ (67 mg, 0.156 mmol) was dissolved in methanol (10 mL). To this solution, cooled in ice bath, a solution of SnCl₂·2H₂O (351 mg, 1.56 mmol) in hydrochloric acid (2 mL) was added dropwise. The reaction mixture was then heated to 85 °C and stirred under reflux for 8 hours. After 8 hours, the mixture was adjusted to neutral pH and extracted multiple times with DCM. The combined organic extracts were dried over anhydrous sodium sulfate. After removal of the solvent under reduced pressure, the residue was purified by column chromatography to afford MeO-D-NH₂ as a tan solid (12 mg, yield 19.3%). ^1^H NMR (400 MHz, Chloroform-*d*) δ 8.21 (d, 1H), 7.51 (d, *J* = 8.3 Hz, 1H), 6.85 (s, 2H), 6.83 (s, 1H), 6.51 (s, 1H), 6.32 (s, 2H), 6.29 (s, 1H), 3.85 (s, 3H), 2.50 (s, 4H), 1.80 (p, *J* = 6.2 Hz, 2H), 1.45 (s, 6H). ^13^C NMR (101 MHz, Chloroform-*d*) δ 159.1, 157.8, 154.4, 148.3, 148.1, 147.9, 132.5, 129.0, 126.8, 122.7, 120.1, 113.7, 113.1, 112.7, 112.3, 111.8, 109.8, 107.8, 101.3, 55.7, 52.5, 29.8, 29.3, 24.9, 21.1. HRMS, m/z: [M+H] + calc. for C_26_H_27_N_2_O_2_ 399.2067, found 399.2078.

**Synthesis of Cl-D-NH₂:** A mixture of Cl-indole (190 mg, 0.984 mmol), compound **1** (100 mg, 0.39 mmol), and potassium acetate (96.4 mg, 0.976 mmol) was placed in a 100 mL round-bottom flask. Acetic acid (5 mL) and acetic anhydride (5 mL) were then added. The reaction mixture was stirred and heated at 80 °C overnight. The following day, the mixture was poured into ice water and neutralized with sodium bicarbonate. The resulting precipitate was collected by filtration and dissolved. The crude product was purified by column chromatography using DCM as the eluent, affording Cl-D-NO₂ as a tan solid (75 mg, 44.5% yield). Cl-D-NO₂ (75 mg, 0.173 mmol) was dissolved in methanol (10 mL). To this solution, cooled in ice bath, a solution of SnCl₂·2H₂O (389.2 mg, 1.73 mmol) in hydrochloric acid (2 mL) was added dropwise. The reaction mixture was then heated to 85 °C and stirred under reflux for 8 hours. After 8 hours, the mixture was adjusted to neutral pH and extracted multiple times with DCM. The combined organic extracts were dried over anhydrous sodium sulfate. After removal of the solvent under reduced pressure, the residue was purified by column chromatography to afford Cl-D-NH₂ as a tan solid (13.2 mg, yield 19.0%). ^1^H NMR (400 MHz, Chloroform-*d*) δ 8.32 (d, 1H), 7.50 (d, *J* = 8.2 Hz, 1H), 7.28 (d, *J* = 2.1 Hz, 1H), 7.24 (d, *J* = 2.1 Hz, 1H), 6.87 (d, *J* = 8.1 Hz, 1H), 6.55 (s, 1H), 6.39 (s, 1H), 6.33 (d, *J* = 7.9 Hz, 1H), 6.27 (d, 1H), 2.51 (s, 4H), 1.81 (p, *J* = 6.2 Hz, 2H), 1.45 (s, 6H). ^13^C NMR (101 MHz, Methanol-*d_4_*) δ 154.4, 152.8, 151.6, 150.5, 147.8, 135.1, 130.1, 127.6, 127.0, 124.9, 124.7, 121.7, 119.1, 112.3, 111.3, 110.3, 109.5, 99.8, 52.4, 29.2, 24.1, 23.4, 20.9. HRMS, m/z: [M+H] + calc. for C_25_H_24_ClN_2_O 403.1572, found 403.1596.

**Synthesis of Br-D-NH₂:** A mixture of Br-indole (185 mg, 0.78 mmol), compound **1** (100 mg, 0.39 mmol), and potassium acetate (76.4 mg, 0.78 mmol) was placed in a 100 mL round-bottom flask. Acetic acid (5 mL) and acetic anhydride (5 mL) were then added. The reaction mixture was stirred and heated at 80 °C overnight. The following day, the reaction mixture was poured into ice water and neutralized with sodium bicarbonate. The resulting precipitate was collected by filtration and dissolved. The crude material was purified by column chromatography using DCM as the eluent, affording Br-D-NO₂ as a tan solid (50 mg, 31.5% yield). Br-D-NO₂ (50 mg, 0.105 mmol) was dissolved in methanol (10 mL). To this solution, cooled in ice bath, a solution of SnCl₂·2H₂O (236.2 mg, 1.05 mmol) in formic acid (2 mL) was added dropwise. The reaction mixture was then heated to 85 °C and stirred under reflux for 4 hours. After 4 hours, the mixture was adjusted to neutral pH and extracted multiple times with DCM. The combined organic extracts were dried over anhydrous sodium sulfate. After removal of the solvent under reduced pressure, the residue was purified by column chromatography to afford Br-D-NH₂ as a tan solid (12.3 mg, yield 26.2%). ^1^H NMR (400 MHz, Chloroform-*d*) δ 8.27 (d, 1H), 7.45 – 7.41 (m, 2H), 7.39 (s, 1H), 6.85 (d, *J* = 8.1 Hz, 1H), 6.49 (s, 1H), 6.36 (s, 1H), 6.32 (d, *J* = 7.2 Hz, 2H), 2.51 (s, 4H), 1.80 (t, *J* = 6.2 Hz, 2H), 1.44 (s, 6H). ^13^C NMR (101 MHz, Methanol-*d_4_*) δ 158.9, 155.7, 154.9, 153.3, 144.9, 138.4, 132.9, 130.9, 128.5, 125.3, 123.9, 117.1, 115.9, 113.3, 112.9, 112.5, 98.5, 50.8, 28.7, 24.6, 24.2, 20.7. HRMS, m/z: [M+H] + calc. for C_25_H_24_BrN_2_O 447.1067, found 447.1079.

**Synthesis of I-D-NH₂:** A mixture of I-indole (222.3 mg, 0.78 mmol), compound **1** (100 mg, 0.39 mmol), and potassium acetate (76.4 mg, 0.78 mmol) was placed in a 100 mL round-bottom flask. Acetic acid (5 mL) and acetic anhydride (5 mL) were then added. The reaction mixture was stirred and heated at 80 °C overnight. The following day, the mixture was poured into ice water and neutralized with sodium bicarbonate. The resulting precipitate was collected by filtration and dissolved. The crude material was purified by column chromatography using DCM as the eluent, affording I-D-NO₂ as a tan solid (74 mg, 36.2% yield). I-D-NO₂ (74 mg, 0.141 mmol) was dissolved in methanol (10 mL). To this solution, cooled in ice bath, a solution of SnCl₂·2H₂O (317.2 mg, 1.41 mmol) in hydrochloric acid (2 mL) was added dropwise. The reaction mixture was then heated to 85 °C and stirred under reflux for 4 hours. After 4 hours, the mixture was adjusted to neutral pH and extracted multiple times with DCM. The combined organic extracts were dried over anhydrous sodium sulfate. After removal of the solvent under reduced pressure, the residue was purified by column chromatography to afford I-D-NH₂ as a tan solid (6.6 mg, yield 9.4%). ^1^H NMR (400 MHz, Chloroform-*d*) δ 8.28 (d, 1H), 7.63 – 7.57 (m, 2H), 7.33 (d, *J* = 8.1 Hz, 1H), 6.85 (d, *J* = 8.1 Hz, 1H), 6.49 (s, 1H), 6.36 (s, 1H), 6.34 – 6.19 (m, 2H), 2.50 (s, 4H), 1.80 (p, *J* = 6.2 Hz, 2H), 1.43 (s, 6H). HRMS, m/z: [M+H] + calc. for C_25_H_24_IN_2_O 495.0928, found 495.0942.

**Synthesis of COOH-D-NH₂:** A mixture of COOH-indole (158.3 mg, 0.78 mmol), compound **1** (100 mg, 0.39 mmol), and potassium acetate (76.4 mg, 0.78 mmol) was placed in a 100 mL round-bottom flask. Acetic acid (5 mL) and acetic anhydride (5 mL) were then added. The reaction mixture was stirred and heated at 80 °C overnight. The following day, the mixture was poured into ice water and the pH was adjusted to a weakly acidic value with sodium bicarbonate. The resulting precipitate was collected by filtration and dissolved. The crude material was purified by column chromatography using DCM as the eluent, affording COOH-D-NO₂ as a tan solid (102 mg, 59.1% yield). COOH-D-NO₂ (100 mg, 0.226 mmol) was dissolved in tetrahydrofuran (THF, 10 mL). To this solution, cooled in ice bath, a solution of SnCl₂·2H₂O (508.5 mg, 2.26 mmol) in hydrochloric acid (2 mL) was added dropwise. The reaction mixture was then heated to 60 °C and stirred under reflux for 4 hours. After 4 hours, the mixture was adjusted to neutral pH and extracted multiple times with DCM. The combined organic extracts were dried over anhydrous sodium sulfate. After removal of the solvent under reduced pressure, the residue was purified by column chromatography to afford COOH-D-NH₂ as a tan solid (32 mg, yield 34.3%). ^1^H NMR (400 MHz, Methanol-*d_4_*) δ 8.41 (d, *J* = 14.9 Hz, 1H), 7.97 (s, 1H), 7.93 (d, *J* = 8.1 Hz, 1H), 7.27 (d, *J* = 8.1 Hz, 1H), 7.07 (d, *J* = 8.3 Hz, 1H), 6.95 (s, 1H), 6.57 (s, 1H), 6.53 (d, *J* = 8.5 Hz, 1H), 6.10 (d, *J* = 14.9 Hz, 1H), 2.58 (t, *J* = 6.0 Hz, 2H), 2.52 (t, *J* = 6.2 Hz, 2H), 1.79 (p, *J* = 6.1 Hz, 2H), 1.46 (s, 6H). ^13^C NMR (101 MHz, Methanol-*d_4_*) δ 182.5, 158.0, 155.5, 152.9, 151.5, 143.00, 138.0, 131.8, 130.2, 128.7, 128.3, 123.9, 123.0, 114.4, 113.2, 112.5, 112.2, 105.3, 98.7, 50.6, 28.7, 24.5, 24.1, 20.7. HRMS, m/z: [M+H] + calc. for C_26_H_25_N_2_O_3_ 413.1860, found 413.1872.

**Synthesis of COOMe-D-NH₂:** COOH-D-NH₂ (20 mg, 0.0485 mmol) was dissolved in methanol (5 mL). The solution was stirred in ice bath for 15 min, followed by the dropwise addition of concentrated sulfuric acid (1 mL). The reaction mixture was then heated to 85 °C and stirred under reflux overnight. The following day, the mixture was adjusted to neutral pH and extracted multiple times with DCM. The combined organic extracts were dried over anhydrous sodium sulfate. After removal of the solvent under reduced pressure, the residue was purified by column chromatography to afford COOMe-D-NH₂ as a tan solid (9 mg, yield 43.5%). ^1^H NMR (400 MHz, Methanol-*d*_4_) δ 8.41 (d, *J* = 15.4 Hz, 1H), 8.03 – 7.99 (m, 2H), 7.44 (d, *J* = 7.3 Hz, 1H), 6.99 (d, *J* = 9.5 Hz, 1H), 6.69 (s, 1H), 6.52 (s, 1H), 6.47 (d, *J* = 8.3 Hz, 1H), 6.26 (d, *J* = 15.4 Hz, 1H), 3.91 (s, 3H), 2.56 (dt, *J* = 13.5, 5.6 Hz, 4H), 1.83 (p, *J* = 6.2 Hz, 2H), 1.48 (s, 6H). ^13^C NMR (101 MHz, Methanol-*d*_4_) δ 167.3, 156.0, 154.8, 151.5, 148.8, 145.1, 136.6, 130.0, 127.5, 125.7, 124.3, 122.5, 117.3, 112.6, 111.1, 109.3, 99.3, 51.6, 51.2, 29.0, 24.1, 23.7, 20.8.

**Synthesis of CN-D-NH₂:** A mixture of CN-indole (143.5 mg, 0.78 mmol), compound **1** (100 mg, 0.39 mmol), and potassium acetate (76.4 mg, 0.78 mmol) was placed in a 100 mL round-bottom flask. Acetic acid (5 mL) and acetic anhydride (5 mL) were then added. The reaction mixture was stirred and heated at 80 °C overnight. The following day, the mixture was poured into ice water and neutralized with sodium bicarbonate. The resulting precipitate was collected by filtration and dissolved. The crude material was purified by column chromatography using DCM as the eluent, affording CN-D-NO₂ as a tan solid (120 mg, 72.7% yield). ^1^H NMR (400 MHz, Chloroform-*d*) δ 8.24 (d, *J* = 16.0 Hz, 1H), 7.88 – 7.81 (m, 2H), 7.68 – 7.61 (m, 2H), 7.56 (s, 1H), 7.12 (d, *J* = 8.2 Hz, 1H), 6.48 (d, *J* = 16.0 Hz, 1H), 6.41 (s, 1H), 2.57 (dt, *J* = 12.2, 5.9 Hz, 4H), 1.86 (t, *J* = 6.3 Hz, 2H), 1.47 (s, 6H).^13^C NMR (101 MHz, Chloroform-*d*) δ 187.3, 158.0, 152.7, 149.8, 147.5, 147.3, 135.2, 134.1, 132.8, 128.3, 125.9, 124.8, 120.9, 120.8, 119.7, 118.6, 117.3, 114.2, 110.3, 107.9, 52.8, 30.1, 24.3, 24.0, 20.4. CN-D-NO₂ (120 mg, 0.283 mmol) was dissolved in methanol (10 mL). To this solution, cooled in ice bath, a solution of SnCl₂·2H₂O (636.75 mg, 2.83 mmol) in hydrochloric acid (2 mL) was added dropwise. The reaction mixture was then heated to 85 °C and stirred under reflux for 4 hours. After 4 hours, the mixture was adjusted to neutral pH and extracted multiple times with DCM. The combined organic extracts were dried over anhydrous sodium sulfate. After removal of the solvent under reduced pressure, the residue was purified by column chromatography to afford CN-D-NH₂ as a tan solid (48 mg, yield 43.1%).^1^H NMR (400 MHz, Chloroform-*d*) δ 8.35 (d, 1H), 7.58 (s, 2H), 7.50 (s, 1H), 6.85 (d, *J* = 9.5 Hz, 1H), 6.44 (s, 1H), 6.38 (s, 1H), 6.33 (d, *J* = 8.1 Hz, 1H), 6.29 (d, 1H), 2.50 (q, *J* = 4.6 Hz, 4H), 1.79 (p, *J* = 6.2 Hz, 2H), 1.44 (s, 6H).^13^C NMR (101 MHz, Chloroform-*d*) δ 187.9, 158.5, 154.3, 152.7, 148.3, 147.0, 135.8, 132.7, 127.7, 125.8, 125.1, 124.4, 120.0, 113.4, 113.0, 110.3, 110.1, 106.7, 101.1, 52.4, 29.7, 24.5, 24.3, 20.9. HRMS, m/z: [M+H] + calc. for C_26_H_24_N_3_O 394.1914, found 394.1928.

Scheme S2. Synthetic route of CN-D-GGT

**Synthesis of CN-D-GGT:** CN-D-NH₂ (25 mg, 0.0636 mmol) and HATU (60.42 mg, 0.159 mmol) were dissolved in DCM (5 mL). To this solution, DIPEA (20.5 mg, 0.159 mmol) dissolved in DCM (1 mL) was added dropwise. The reaction mixture was stirred under ice bath. After 30 min, compound **2** (38.5 mg, 0.127 mmol) was added. The reaction was then allowed to room temperature and stirred overnight. On the following day, the reaction mixture was washed twice with water and then concentrated under reduced pressure to obtain the crude product. The crude product was dissolved in DCM (4 mL), followed by dropwise addition of trifluoroacetic acid (TFA, 1 mL). The mixture was stirred overnight at room temperature. The next day, the solvent was removed under reduced pressure, and the residue was purified by preparative thin-layer chromatography (PTLC) to afford CN-D-GGT as a tan solid (6.5 mg). ^1^H NMR (400 MHz, Methanol-*d*_4_) *δ* 8.41 (d, 1H), 7.75 (s, 1H), 7.71 – 7.63 (m, 2H), 7.56 (d, *J* = 8.1 Hz, 1H), 7.10 (s, 2H), 6.57 (s, 1H), 6.39 (d, *J* = 15.7 Hz, 1H), 3.66 (d, *J* = 6.2 Hz, 1H), 2.65 (t, *J* = 7.2 Hz, 2H), 2.57 (d, *J* = 7.5 Hz, 2H), 2.55 (s, 2H), 2.23 – 2.16 (m, 2H), 1.83 (t, *J* = 6.2 Hz, 2H), 1.46 (s, 6H).^13^C NMR (101 MHz, Methanol-*d*_4_) *δ* 190.1, 173.4, 158.6, 154.5, 153.8, 148.4, 141.2, 137.4, 134.0, 130.3, 127.6, 126.5, 125.4, 125.0, 120.6, 120.5, 119.4, 115.9, 114.1, 112.5, 108.5, 107.3, 34.0, 31.9, 30.7, 27.6, 25.4, 24.2, 22.0, 9.2. HRMS, m/z: [M+H] + calc. for C_31_H_31_N_4_O_4_ 523.2340, found 523.2354.

**Supplementary Figures**

**Figure S1**. Frontier molecular orbital distributions and the major electronic transition characteristics of the molecule before and after protonation. Upon protonation, the LUMO energy level is markedly lowered and the S_0_→S_1_ transition energy decreases, indicating an enhanced electron-accepting character and a strengthened intramolecular charge transfer process.

**Figure S2.** (a) Absorption spectra and fluorescence emission spectra (b) of D-NH_2_ (5 μM) in varying pH buffer. (c) Absorption spectra and fluorescence emission spectra (d) of Cl-D-NH_2_ (5 μM) in varying pH buffer. (e) Absorption spectra and fluorescence emission spectra (f) of Br-D-NH_2_ (5 μM) in varying pH buffer. (g) Absorption spectra and fluorescence emission spectra (h) of I-D-NH_2_ (5 μM) in varying pH buffer. (i) Absorption spectra and fluorescence emission spectra (j) of COOH-D-NH_2_ (5 μM) in varying pH buffer. (k) Absorption spectra and fluorescence emission spectra (l) of H_3_CO-D-NH_2_ (5 μM) in varying pH buffer. (m) Absorption spectra and fluorescence emission spectra (n) of COOMe-D-NH_2_ (5 μM) in varying pH buffer. (o) Absorption spectra and fluorescence emission spectra (p) of Me-D-NH_2_ (5 μM) in varying pH buffer. The test buffer was conducted in PBS/EtOH = 9/1, v/v, 20 mM, pH = 5, 6.5, or 7.

**Figure S3.** (a) Relative changes in absorption and fluorescence intensity of Dx-NH_2_ synthesized from indole substituted with 8 functional groups (methyl-, methoxy-, cyano-, carboxy-, ester-, and halogen groups) at pH 5 and 6.5. (b) Relative changes in absorption and fluorescence intensity of Dx-NH_2_ synthesized from indole substituted with 8 functional groups (methyl-, methoxy-, cyano-, carboxy-, ester-, and halogen groups) at pH 5 and 7.5.

**Figure S4.** Cell survival of cells treated with CN-D-NH_2_ (5, 10, 20, 40 µM, 24 h) in HepG2 cells. Error bars are presented as mean ± standard deviation (SD), n = 5.

**Figure S5.** (a) Real time imaging of HepG2 cells co cultured with CN-D-NH_2_ over time. (b) Quantification of fluorescence intensity from panel a. Scale bars, 20 μm. Error bars are presented as mean ± standard deviation (SD), n = 5.

**Figure S6.** (a) Absorption spectra of CN-D-GGT (5 μM) incubated with GGT (0-200 U/L) at pH 6.5. (b) PA. intensity of CN-D-GGT (5 μM) incubated with GGT (0-60 U/L) at pH 6.5.

**Figure S7.** (a) Surface drawing of CN-D-GGT at the binding cleft of GGT (PDB ID: 4ZBK). (b) Surface drawing of CN-D-GGT [H+] at the binding cleft of GGT. Molecular docking results of CN-D-GGT and GGT. The yellow line represents hydrogen bond; The red line represents the distance between the active center and the cutting position.

**Figure S8.** Cell survival of cells treated with CN-D-GGT (2, 4, 6, 10, 15, 20 µM, 24 h) in HepG2 cells. Error bars are presented as mean ± standard deviation (SD), n = 3.

**Figure S9.** (a) Representative photoacoustic images of cancer cells (HepG2 and HeLa) and normal cells (LX-2 and HEK293T) after 1 h of incubation with CN-D-GGT (5 µM). (b) Quantification of the photoacoustic signal intensity in panel a. Error bars are presented as mean ± standard deviation (SD), n = 3. Statistical significance is indicated by *p < 0.05, **p < 0.01. ns, not significant (t-test).

**Figure S10.** Representative fluorescence images of CN-D-GGT with Lyso-Tracker Green in HepG2 cells. Scale bar: 20 µm.

**Figure S11.** (a) Fluorescence images and (b) photoacoustic images of HepG2 cells treated with DON (200 µM, 2 h), followed by CN-D-GGT (5 µM). Error bars are presented as mean ± standard deviation (SD), n = 3. Scale bar: 20 µm. Statistical significance is indicated by ***p < 0.001, *p < 0.05, based on the t-test.

**Figure S12.** (a) Representative fluorescence images of normal and tumor tissues treated with CN-D-GGT. (b) Quantification of fluorescence intensity from panel a. Scale bars, 20 μm. Error bars are presented as mean ± standard deviation (SD), n = 5. Statistical significance is indicated by ****p < 0.0001, based on the t-test.

**Figure S13.** Fluorescence and photoacoustic images of three tissue models before and after in situ injection of CN-D-GGT (50 μM, 25 μL).

**Figure S14.** Representative images of H&E-stained slices of major organs from the mice in each group after intravenous injection of CN-D-GGT or PBS as control for 24 h.

**Figure S15.** a, Representative fluorescence and photoacoustic images before and after intratumoral injection of CN-D-GGT; b, Representative fluorescence and photoacoustic images before and after intratumoral injection of CN-D-GGT after tumor pretreatment with DON (1 mM, 2 h). c, Quantification of fluorescence intensity from panel a. d, Quantification of fluorescence intensity from panel b.

**Figure S16.** Representative fluorescence images of clinical small volume (about 0.5×0.2×0.2 cm) HCC samples. HCC samples were soaked in CN-D-GGT (50 µM) for 10 min before fluorescence imaging.

**Figure S17.** The ^1^H-NMR spectrum of D-NH_2_.

**Figure S18.** The ^13^C-NMR spectrum of D-NH_2_.

**Figure S19.** The HRMS of D-NH_2_.

**Figure S20.** The ^1^H-NMR spectrum of Me-D-NH_2_.

**Figure S21.** The ^13^C-NMR spectrum of Me-D-NH_2_.

**Figure S22.** The HRMS of Me-D-NH_2_.

**Figure S23.** The ^1^H-NMR spectrum of Cl-D-NH_2_.

 **Figure S24.** The ^13^C-NMR spectrum of Cl-D-NH_2_.

**Figure S25.** The HRMS of Cl-D-NH_2_.

**Figure S26.** The ^1^H-NMR spectrum of Br-D-NH_2_.

**Figure S27.** The ^13^C-NMR spectrum of Br-D-NH_2_.

**Figure S28.** The HRMS of Br-D-NH_2_.

**Figure S29.** The ^1^H-NMR spectrum of I-D-NH_2_.

**Figure S30.** The HRMS of I-D-NH_2_.

**Figure S31.** The ^1^H-NMR spectrum of H_3_CO-D-NH_2_.

**Figure S32.** The ^13^C-NMR spectrum of H_3_CO-D-NH_2_.

**Figure S33.** The HRMS of H_3_CO-D-NH_2_.

**Figure S34.** The ^1^H-NMR spectrum of COOH-D-NH_2_.

**Figure S35.** The ^1^H-NMR spectrum of COOH-D-NH_2_.

**Figure S36.** The HRMS of COOH-D-NH_2_.

**Figure S37.** The ^1^H-NMR spectrum of COOMe-D-NH_2_.

**Figure S38.** The ^13^C-NMR spectrum of COOMe-D-NH_2_.

**Figure S39.** The ^1^H-NMR spectrum of CN-D-NH_2_.

**Figure S40.** The ^13^C-NMR spectrum of CN-D-NH_2_.

**Figure S41.** The HRMS of CN-D-NH_2_.

 **Figure S42.** The ^1^H-NMR spectrum of CN-D-GGT.

**Figure S43.** The ^13^C-NMR spectrum of CN-D-GGT.

**Figure S44.** The HRMS of CN-D-GGT.

**References**

1 Z. Yang, F. Chen, Y. He, N. Yang, Q.-H. Fan, *Angew. Chem. Int. Ed.* **2016**, *55*, 13863.

2 X. Zhang, X. Li, W. Shi and H. Ma, *Chem. Commun.*, **2021**, 57, 8174-8177
